# Selection of haplotype variables from a high-density marker map for genomic prediction

**DOI:** 10.1186/s12711-015-0143-3

**Published:** 2015-08-01

**Authors:** Beatriz CD Cuyabano, Guosheng Su, Mogens S. Lund

**Affiliations:** Center for Quantitative Genetics and Genomics, Department of Molecular Biology and Genetics, Aarhus University, Aarhus, Denmark

## Abstract

**Background:**

Using haplotype blocks as predictors rather than individual single nucleotide polymorphisms (SNPs) may improve genomic predictions, since haplotypes are in stronger linkage disequilibrium with the quantitative trait loci than are individual SNPs. It has also been hypothesized that an appropriate selection of a subset of haplotype blocks can result in similar or better predictive ability than when using the whole set of haplotype blocks. This study investigated genomic prediction using a set of haplotype blocks that contained the SNPs with large effects estimated from an individual SNP prediction model. We analyzed protein yield, fertility and mastitis of Nordic Holstein cattle, and used high-density markers (about 770*k* SNPs). To reach an optimum number of haplotype variables for genomic prediction, predictions were performed using subsets of haplotype blocks that contained a range of 1000 to 50 000 main SNPs.

**Results:**

The use of haplotype blocks improved the prediction reliabilities, even when selection focused on only a group of haplotype blocks. In this case, the use of haplotype blocks that contained the 20 000 to 50 000 SNPs with the highest effect was sufficient to outperform the model that used all individual SNPs as predictors (up to 1.3 % improvement in prediction reliability for mastitis, compared to individual SNP approach), and the achieved reliabilities were similar to those using all haplotype blocks available in the genome data (from 0.6 % lower to 0.8 % higher reliability).

**Conclusions:**

Haplotype blocks used as predictors can improve the reliability of genomic prediction compared to the individual SNP model. Furthermore, the use of a subset of haplotype blocks that contains the main SNP effects from genomic data could be a feasible approach to genomic prediction in dairy cattle, given an increase in density of genotype data available. The predictive ability of the models that use a subset of haplotype blocks was similar to that obtained using either all haplotype blocks or all individual SNPs, with the benefit of having a much lower computational demand.

## Background

Since genomic selection methods were introduced [1], single nucleotide polymorphisms (SNPs) are usually used to perform genomic prediction, each as an individual explanatory variable independent from each other. An alternative is to use haplotypes as explanatory variables in genomic prediction. The main benefit of using haplotypes for genomic prediction is that haplotypes are expected to be in higher linkage disequilibrium (LD) with the quantitative trait loci (QTL) than individual SNPs are, meaning that an individual marker effect is not necessarily the best predictor of a QTL effect [2]. Hence, haplotypes used as predictors to estimate breeding values are expected to improve results.

A previous study based on simulated data showed that the use of haplotypes leads to higher prediction reliabilities than individual marker predictors [3]. Using haplotype blocks (haploblocks) based on LD, from a high-density (HD) marker data in the Nordic Holstein population, reliability of genomic prediction for economically important traits was increased by 3 % when compared to predictions using individual SNPs [4]. Based on these considerations, it is reasonable to assume that haploblocks based on LD should be good explanatory variables for genomic prediction. Assuming a correct marker map, one of the advantages of using haploblocks based on LD is the non-fixed number of SNPs in a haploblock which allowed the grouping of tightly linked adjacent SNPs. Therefore, it efficiently limits the number of “alleles” per haploblock. However, it is clear that the construction of haploblocks based on LD requires an appropriate choice of LD measure and a minimum threshold of LD between markers. The choices that were applied in our work are described in the Methods section. One interesting alternative to LD-based haploblocks are genomic prediction methods based on identity-by-descent (IBD) relationships [5, 6]; this alternative benefits from linkage analysis over the genomic data. However, prediction accuracies of breeding values depend strongly on the number of phenotyped and genotyped relatives within the population [6]. Besides, this method using IBD relationships aims at decreasing marker density to reduce genotyping cost, whereas the method based on haploblocks in the current study aims at reducing prediction variables from HD marker data. Thus, the LD-based haploblocks were preferred in this study.

Genomic prediction using a set of appropriately selected haploblocks is expected to achieve higher prediction accuracy while reducing the amount of predictor variables in prediction models. A recent study showed that better predictions in dairy cattle traits can be obtained by using a set of haploblocks with a fixed size (number of SNPs) [7]. Our hypothesis is that using haploblocks that contain the main SNP effects (*i.e.* the SNPs with the highest absolute effect estimated using the models which estimated effects of all individual SNPs simultaneously, and the haploblocks containing these SNPs are referred to as QTL-haploblocks hereafter) can improve genomic prediction. By using QTL-haploblocks, it is possible to identify the parts of the genome that strongly influence the predictions of livestock traits. In addition, a large proportion of haploblocks may have no effect on a trait, and removing them as covariates may reduce the noise in genomic prediction models. The idea of a QTL-haploblock approach is similar to marker-assisted selection (MAS). However, MAS performs predictions using only a few genetic markers with a significant effect, that was previously estimated by a model including all individual SNPs [8, 9], while the proposed QTL-haploblock approach uses genome-wide dense markers and allows a large number of markers into the model, not necessarily all with a significant effect.

Using pre-selected haploblocks for genomic prediction is an important area of research, especially when considering the availability of increasingly denser SNP chips. Reliability of genomic prediction for a trait is expected to be improved by identifying the most influential haploblocks for this trait to be included in the prediction model. In addition, genomic prediction models including a selected group of haploblocks will reduce computing time considerably, compared to models using all haploblocks. This is not necessarily relevant when dealing with moderate-density marker data but plays an important role when dealing with high-density marker data, and is more important when using whole-genome sequence data.

Therefore, this study compared genomic predictions using an individual SNP approach, a haploblock approach with all available haploblocks, and a haploblock approach using a set of haploblocks that contained the main SNPs. The analyses were performed using data from the Nordic Holstein population. The key objective of this work was to assess prediction reliability obtained by using QTL-haploblocks as covariates, and to compare them to those achieved when using all individual SNPs or all haploblocks from a high-density marker chip.

## Methods

### Marker and phenotypic data

The genomic prediction models performed in this study were based on marker and phenotypic data from the Nordic Holstein population. The original marker data was obtained from a 54*k* SNP chip and then imputed to high-density (HD) data of 777k SNPs (Ilumina BovineHD BeadChip [10]), by applying the Beagle package [11], using 557 HD genotyped reference bulls from the EuroGenomics project [12]. The imputed data was then edited by removing SNPs with a minor allele frequency (MAF) less than 0.01 and also SNPs that were in complete LD with adjacent ones [13]. After editing, the final marker data set included 492 057 SNPs for 5214 bulls.

The phenotypic values to perform genomic prediction for protein yield, fertility and mastitis were pseudo-observations in the form of deregressed proofs (DRP), obtained from the estimated breeding values (EBV) and effective daughter contributions [14–16]. All three traits are index traits. The EBV for protein yield and mastitis were composed of EBV for each parity. The EBV for fertility comprised the EBV for interval from calving to first insemination, interval from first to last inseminations, and number of inseminations (heifer and cow separately, pooled over parities for cow). The DRP of all the animals (both training and validation) were derived from the EBV of the official evaluation in August 2010 by the Nordic Cattle Genetic Evaluation (NAV).

To validate the predictive ability of the prediction models, the marker and phenotypic data sets were divided into training and test subsets by the cut-off birth date of bulls on October 1, 2001. The size of training and test data sets are in Table 1, as well as trait reliabilities for these populations.

**Table 1:** Size, reliability of deregressed proofs and heritability (*h* ^2^) of the training and test data sets used for genomic prediction

	Protein (*h* ^2^=0.39)			Fertility (*h* ^2^=0.04)			Mastitis (*h* ^2^=0.04)	
	pop. size	$r_{\textit {DRP}}^{2}$		pop. size	$r_{\textit {DRP}}^{2}$		pop. size	$r_{\textit {DRP}}^{2}$
Train	3003	0.940		3037	0.683		3006	0.824
Test	1395	0.924		1378	0.608		1491	0.750
Total	4398	0.935		4415	0.659		4497	0.800

### Animal ethics

The phenotypic data were obtained from routine records of dairy cattle farms. Genotyped animals used in this work were progeny-tested bulls, and the semen samples for genotyping were obtained from routine bull semen collection. Therefore, no ethical approval was necessary.

### Genomic prediction covariates

Genomic predictions were performed using individual SNPs and haploblocks. Haploblocks were built based on LD and then selected according to specific criteria. In this section, first we briefly describe the construction of haploblocks and then their selection for genomic prediction.

There are three common pairwise LD measures, *D*, *r*^2^ and *D*^′^ [17, 18]. In this study, *D*^′^ was chosen to define haploblocks according to a previous study [19], and due to the fact that it depends less on individual allele frequencies than *D*. In addition, a pilot study was performed to compare predictions using haploblocks that were defined using *r*^2^ and *D*^′^, and no difference on predictive ability was observed. Because the use of *r*^2^ led to many more haploblocks, *D*^′^ was finally chosen as the most adequate LD measure to build haploblocks.

Following our previous study [4], a haploblock was defined as a group of adjacent SNPs such that the LD between any pair of SNPs in this group satisfies |*D*^′^|≥0.45. This threshold of 0.45 was considered as optimal, considering the prediction reliability, to predict genomic breeding values for the three traits of interest using all the haploblocks built from the HD marker data [4].

Using this LD criterion to define the haploblocks resulted in a total of 76 062 haploblocks. Because haploblocks are “multi-allelic” it summed up a total of 368 709 haploblock variables. The number of SNPs in a haploblock ranged from 1 to 78, with a mean of 6. The number of variants within a haploblock ranged from 1 to 16, with a mean number of 5 [4]. Haploblocks that had only one variant were excluded.

Selection of haploblocks was based on the estimated SNP effects obtained from prediction models using either Bayesian best linear unbiased prediction (Bayesian BLUP) or a Bayesian mixture model, based on the training dataset. Detailed description of the models is provided in the next section, entitled Genomic prediction models. For each trait, the absolute values of the estimated SNP effects were ranked. Then, a determined number *k* of SNPs with the highest effects was defined. Finally, the haploblocks containing those SNPs were selected to perform genomic prediction. The number *k* of SNP effects used to select the haploblocks varied from 1000 to 50 000. In the following, the haploblocks selected according to the SNP effects estimated from the training dataset will be referred to as QTL-haploblocks.

Haploblocks that were selected by SNP effects estimated by the Bayesian BLUP model were used for genomic prediction using the Bayesian BLUP model. Analogously, haploblocks that were selected by SNP effects estimated by the Bayesian mixture model were used for genomic prediction using the Bayesian mixture model. Because the estimates of SNP effects differed according to trait and model, the ranking of SNP effects differed as well, thus the number of main SNPs within a haploblock varied. This resulted in different selected haploblocks for each trait, and the number of haploblocks, used to perform genomic prediction.

In order to confirm that genomic prediction using QTL-haploblocks obtains more accurate results than selecting the haploblocks randomly, protein yield was analysed using haploblocks containing 1000 to 50 000 randomly selected SNPs. This procedure was repeated 10 times, and the reliabilities of the predictions were compared to the reliabilities of predictions obtained using QTL-haploblocks.

### Genomic prediction models

For the three traits mentioned previously, genomic predictions were performed using a Bayesian BLUP or a Bayesian mixture model, both including the QTL-haploblocks effect and a polygenic effect. The two models used a Bayesian algorithm and were performed using the BayZ package [20], running a single Markov chain Monte Carlo (MCMC) with a length of 50 000, of which the first 20 000 cycles were taken as the burn-in of the chain. Estimates were assessed by the posterior means of the non-discarded 30 000 cycles. Convergence and length of MCMC were monitored by graphical inspection of the dispersion parameter in the models and the correlation between the genomic estimated breeding values (GEBV) from two separate rounds in a pilot study.

#### Bayesian BLUP model

The Bayesian BLUP model is defined by the equation

(1) $$ \mathbf{y} = \boldsymbol{1}\mu + \mathbf{M} \mathbf{g} + \mathbf{Z} \mathbf{a} + \boldsymbol{\epsilon},   $$

where **y** represents the vector containing the DRP of training bulls, *μ* a general mean, **M** the haploblock matrix, **g** the vector of additive haploblock effects, **Z** the incidence matrix linking **a** to **y**, **a** the vector of residual polygenic additive genetic effects and ***ε*** the vector of random errors of the model. It is assumed that the distributions are as follows,

(2) $$\begin{array}{@{}rcl@{}} \begin{array}{l} \mathbf{g} \sim N\left(\boldsymbol{0},\mathbf{I}{\sigma_{g}^{2}}\right) \\ \mathbf{a} \sim N\left(\boldsymbol{0},\mathbf{A}{\sigma_{a}^{2}}\right) \\ \boldsymbol{\epsilon} \sim N\left(\boldsymbol{0}, \mathbf{D} \sigma_{\epsilon}^{2}\right) \\ \mu, {\sigma_{g}^{2}}, {\sigma_{a}^{2}}, \sigma_{\epsilon}^{2} \sim Uniform, \end{array}  \end{array} $$

where **A** is the genetic relationship matrix constructed according to pedigree, **D** is a diagonal matrix with *d*_*ii*_=1/*w*_*i*_ and $w_{i} = r_{\textit {DRPi}}^{2}/(1 - r_{\textit {DRPi}}^{2})$ [16, 21]. Furthermore, *w*_*i*_ is a weighting factor accounting for heterogeneous residual variances due to differences in $r_{\textit {DRPi}}^{2}$, the *i*−*th* DRP’s reliability [22]. The prior uniform distributions were always improper, care was taken to ensure that the overall mean was within the real values and the variances were positive real values.

Taking into account that each haploblock may have more than two variants, matrix ***M*** may have more than one column for each haploblock and had dimension *n*×*q* (*n*= number of animals, *q*= total number of haploblock variables).

#### Bayesian mixture model

The Bayesian mixture model is defined by the same equation and variables as the Bayesian BLUP model but differs in the assumed distribution of **g**, the additive haploblock effects, given by

(3) $$ \mathbf{g} \sim {\sum\nolimits}_{k=1}^{4} \pi_{k}N\left(\boldsymbol{0}, \mathbf{I}\sigma_{\pi_{k}}^{2}\right).   $$

This Bayesian mixture model [23] is an extended version of simpler ones [24, 25], and intends to facilitate the mixing of the MCMC on the high-density marker data. The mixing proportions *π*_*k*_ were fixed at *π*_1_=0.889, *π*_2_=0.1, *π*_3_=0.01 as *π*_4_=0.001, and the variances were estimated under the constraint $\sigma _{\pi _{1}}^{2} < \sigma _{\pi _{2}}^{2} < \sigma _{\pi _{3}}^{2} < \sigma _{\pi _{4}}^{2}$ assuming a non-informative prior uniform distribution.

### Evaluation of prediction models

GEBV obtained from the prediction models were calculated as $GEBV_{i} = \sum _{j}m_{\textit {ij}}\hat {g}_{j} + \hat {a}_{i}$, the performance of each model was assessed by the estimated reliability of GEBV, *r*^2^ and the bias of GEBV. The bias was assessed as *b*−1, where *b* is the regression coefficient *b* of DRP on the GEBV [22].

The reliability of the prediction for breeding values was obtained as the squared correlation between DRP and GEBV of individuals in the test population corrected for the average reliability of DRP of the test animals $\left (r^{2}_{\textit {DRP}}\right)$ [16]. Thus, the average reliability of GEBV in the test population was calculated as,

(4) $$ r^{2} = \frac{Cor^{2}(DRP,GEBV)}{r^{2}_{DRP}}.   $$

One of the objectives of this study was to test if fitting a group of selected haploblocks performed as well or better than fitting all haploblocks from the marker data. Thus, reliabilities of models with selected haploblocks were compared to the reliability of the model using all haploblocks with the Hotelling-Williams’ t-test [26, 27]. Testing whether *r*^2^[ prediction 1]=*r*^2^[ prediction 2] is equivalent to testing whether *Cor*(*DRP*,*GEBV*[prediction 1])=*Cor*(*DRP*,*GEBV*[ prediction 2]). Let *ρ*_*drp*,*i*_=*Cor*(*DRP*,*GEBV*[prediction *i*]) and *ρ*_*ij*_=*Cor*(*GEBV*[ prediction *i*],*GEBV*[ prediction *j*]), the statistic to test whether *H*_0_:*ρ*_*drp*,*i*_=*ρ*_*drp*,*j*_ is true versus *H*_1_:*ρ*_*drp*,*i*_≠*ρ*_*drp*,*j*_, is given by,

(5) $$  T = \! \frac{(r_{drp,i} - r_{drp,j})\sqrt{(n-3)(1+r_{ij})/2|\mathbf{R}|}}{\sqrt{1 + (n-3)(r_{drp,i}+r_{drp,j})^{2}(1-r_{ij})^{3}/[8(n-1)|\mathbf{R}|]}},   $$

where *r*_∗∗_ refers to the observed values of the correlations *ρ*_∗∗_, as described above, *n* the number of observations and |**R**| is the determinant of the correlation matrix **R** for *DRP* and *GEBV* for models *i* and *j*. If the null hypothesis is true, then *T*∼*t*_*n*−3_, hence if |*T*|≥*t*_0_, such that *P*(|*T*|≥*t*_0_)≤*α*, then *H*_0_ is rejected and it is considered that *ρ*_*drp*,*i*_≠*ρ*_*drp*,*j*_ at a significance level *α*. *T* statistics and their associated p-values were calculated using R [28].

## Results

Table 2 presents the number of QTL-haploblocks selected for each trait and for both statistical models used for genomic prediction. Because the selection of haploblocks was based on the SNP effects obtained from two models that included all individual SNPs, the haploblocks selected differed by trait and model. It can be observed that when using up to 10 000 main SNPs to select QTL-haploblocks, the number of haploblocks did not differ much from the number of main SNPs. Between 20 000 and 50 000 this difference was more pronounced, which means that the first 10 000 SNPs with the highest effects were located in different haploblocks, while thereafter more than one main SNP fell in the same haploblock.

**Table 2:** Total number of selected haploblocks to be used in the prediction models of the three traits using Bayesian BLUP or mixture models, according to number of main SNP effects

Main	Protein			Fertility			Mastitis	
SNPs ^*†*^	BLUP	4 mixture		BLUP	4 mixture		BLUP	4 mixture
1000	987	988		989	994		991	985
2000	1951	1951		1952	1965		1954	1949
3000	2893	2903		2906	2928		2913	2897
4000	3828	3845		3848	3870		3843	3827
5000	4753	4763		4763	4771		4761	4730
6000	5643	5661		5658	5669		5646	5627
7000	6538	6541		6529	6545		6543	6501
8000	7398	7411		7378	7388		7411	7354
9000	8219	8264		8231	8241		8256	8218
10 000	9043	9101		9061	9078		9067	9037
20 000	16 577	16 686		16 641	16 660		16 584	16 553
30 000	22 958	23 015		23 016	23 017		22 974	22 866
40 000	28 386	28 531		28 468	28 436		28 380	28 349
50 000	33 120	33 189		33 276	33 110		33 092	33 059
492 057	76 062	76 062		76 062	76 062		76 062	76 062

^*†*^number of highest (absolute) SNP effects used to select haploblocks

Table 3 shows the total number of haploblock variables. Since haploblocks are “multi-allelic”, the numbers in Table 3 represent the sum of these alleles, for the selected haploblocks. These were the total number of covariates used in the genomic prediction models.

**Table 3:** Total number of haploblock variables to be used in the prediction models of the three traits using Bayesian BLUP or mixture models, according to number of main SNP effects

Main	Protein			Fertility			Mastitis	
SNPs ^*†*^	BLUP	4 mixture		BLUP	4 mixture		BLUP	4 mixture
1000	5701	5761		5824	6007		5815	5816
2000	11 479	11 387		11 478	11 657		11 514	11 475
3000	17 012	16 854		17 016	17 258		17 000	17 081
4000	22 464	22 311		22 494	22 720		22 378	22 603
5000	27 777	27 741		27 790	27 976		27 754	27 775
6000	32 956	32 941		33 046	33 232		32 910	32 957
7000	38 223	38 024		38 097	38 222		38 197	37 946
8000	43 166	43 099		42 938	43 121		43 265	42 893
9000	47 838	48 044		47 966	48 076		48 101	47 879
10 000	52 553	52 938		52 697	52 783		52 769	52 573
20 000	95 234	95 882		95 669	95 710		95 459	95 055
30 000	130 572	130 865		131 045	130 969		130 735	129 994
40 000	160 108	160 600		160 355	160 195		160 000	159 680
50 000	185 225	185 603		185 839	185 000		184 935	184 661
492 057	368 709	368 709		368 709	368 709		368 709	368 709

^*†*^number of highest (absolute) SNP effects used to select haploblocks

Table 4 presents the prediction reliabilities and bias for the three traits using each prediction model. The row with 492 057 main SNPs corresponds in fact to the haploblock approach using all haploblocks (full haploblocks model) and the last row is the SNP approach. These results were the basis for the comparison of predictions using selected QTL-haploblocks. In this table, we observe that prediction reliabilities using QTL-haploblocks selected by 20 000 to 50 000 main SNPs were greater (up to 1.3 % observed in the prediction of mastitis) than those achieved by using the individual SNP approach, in most cases. An exception was observed for the prediction of fertility using 40 000 main SNPs, for which case the prediction reliability was 0.1 % lower than that achieved by the individual SNP approach. In addition, the differences between the bias obtained by the QTL-haploblock model and the full haploblock model were very small for the three traits. The observed biases, measured as the deviation of the regression coefficients of DRP on GEBV, to 1, was between 0.002 and 0.181 among the three traits.

**Table 4:** Results^a^ of genomic prediction of the three traits using Bayesian BLUP or mixture models, according to number of main SNP effects

Main	Protein			Fertility			Mastitis	
SNPs ^*†*^	BLUP	4 mixture		BLUP	4 mixture		BLUP	4 mixture
1000	0.347 (-0.124)	0.396 (-0.181)		0.356 (0.060)	0.334 (0.040)		0.319 (0.028)	0.318 (0.085)
2000	0.376 (-0.115)	0.409 (-0.174)		0.364 (0.055)	0.333 (0.056)		0.333 (0.026)	0.346 (0.054)
3000	0.400 (-0.107)	0.413 (-0.171)		0.348 (0.016)	0.359 (0.008)		0.345 (0.009)	0.359 (0.033)
4000	0.410 (-0.107)	0.422 (-0.164)		0.359 (0.026)	0.370 (0.012)		0.348 (0.008)	0.368 (0.019)
5000	0.417 (-0.102)	0.426 (-0.160)		0.368 (0.035)	0.375 (0.022)		0.358 (0.002)	0.371 (0.012)
6000	0.422 (-0.102)	0.430 (-0.161)		0.367 (0.026)	0.376 (0.028)		0.358 (0.009)	0.373 (0.016)
7000	0.423 (-0.108)	0.435 (-0.155)		0.369 (0.031)	0.378 (0.034)		0.363 (0.006)	0.374 (0.006)
8000	0.426 (-0.106)	0.437 (-0.143)		0.373 (0.041)	0.384 (0.044)		0.366 (0.003)	0.378 (0.002)
9000	0.425 (-0.103)	0.440 (-0.136)		0.373 (0.035)	0.384 (0.037)		0.368 (0.008)	0.376 (0.009)
10 000	0.428 (-0.100)	0.441 (-0.137)		0.376 (0.040)	0.389 (0.054)		0.369 (0.006)	0.375 (0.011)
20 000	0.427 (-0.101)	0.446 (-0.122)		0.384 (0.048)	0.384 (0.033)		0.372 (0.012)	0.382 (0.003)
30 000	0.427 (-0.111)	0.448 (-0.116)		0.384 (0.048)	0.388 (0.048)		0.372 (0.018)	0.386 (0.006)
40 000	0.427 (-0.108)	0.446 (-0.114)		0.383 (0.043)	0.390 (0.043)		0.373 (0.008)	0.384 (0.003)
50 000	0.424 (-0.121)	0.447 (-0.115)		0.385 (0.051)	0.387 (0.044)		0.375 (0.008)	0.383 (0.006)
492 057	0.429 (-0.120)	0.447 (-0.127)		0.389 (0.057)	0.390 (0.057)		0.372 (0.005)	0.378 (0.005)
Indiv. SNP	0.423 (-0.122)	0.439 (-0.122)		0.384 (0.059)	0.383 (0.048)		0.368 (0.069)	0.373 (0.026)
approach								

^a^values displayed as: reliability (prediction bias)

^*†*^number of highest (absolute) SNP effects used to select haploblocks

**Fig. 1:**
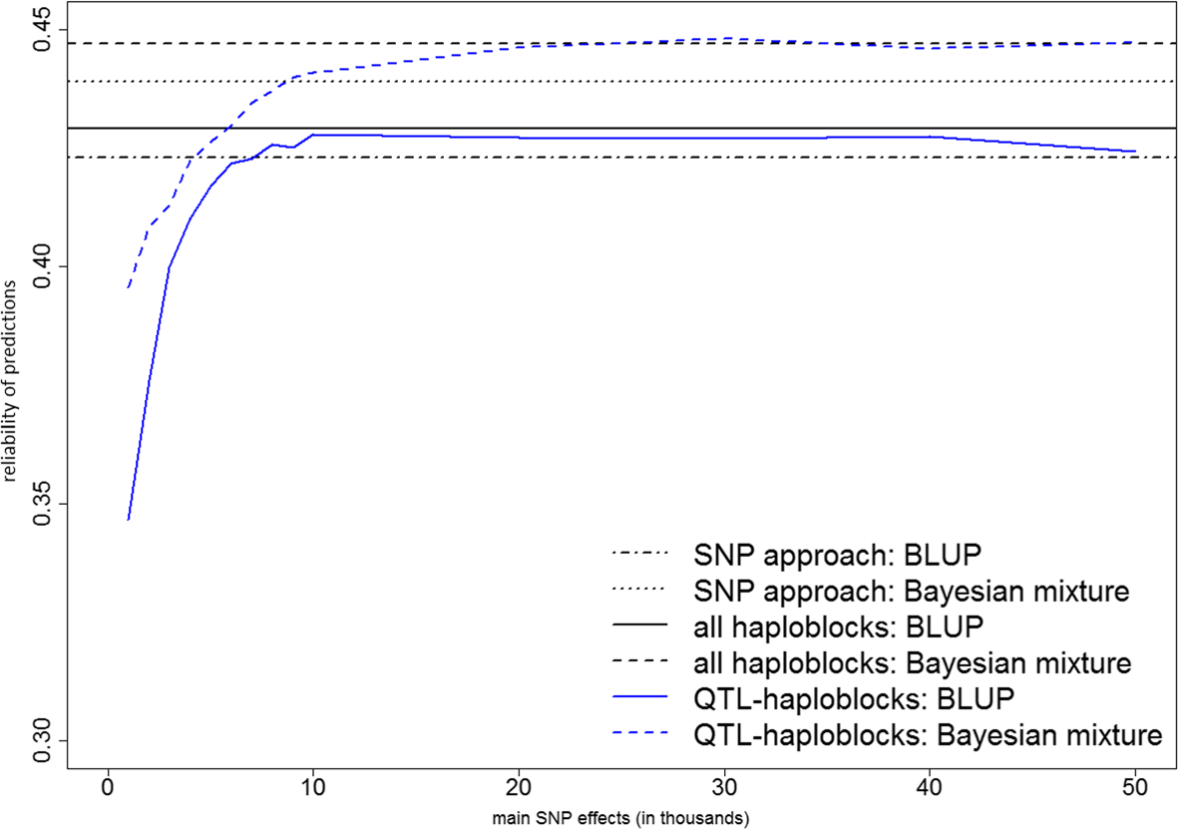
Prediction reliabilities obtained using models with QTL-haploblocks as covariates for protein yield. The values on the x-axis are the number of main SNPs used to select QTL-haploblocks to perform genomic prediction, and the y-axis indicates the reliability of predictions

**Fig. 2:**
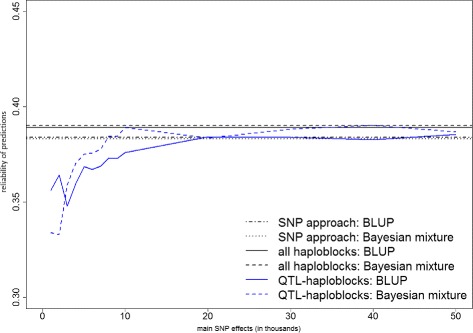
Prediction reliabilities obtained using models with QTL-haploblocks as covariates for fertility. The values on the x-axis are the number of main SNPs used to select QTL-haploblocks to perform genomic prediction, and the y-axis indicates the reliability of predictions

**Fig. 3:**
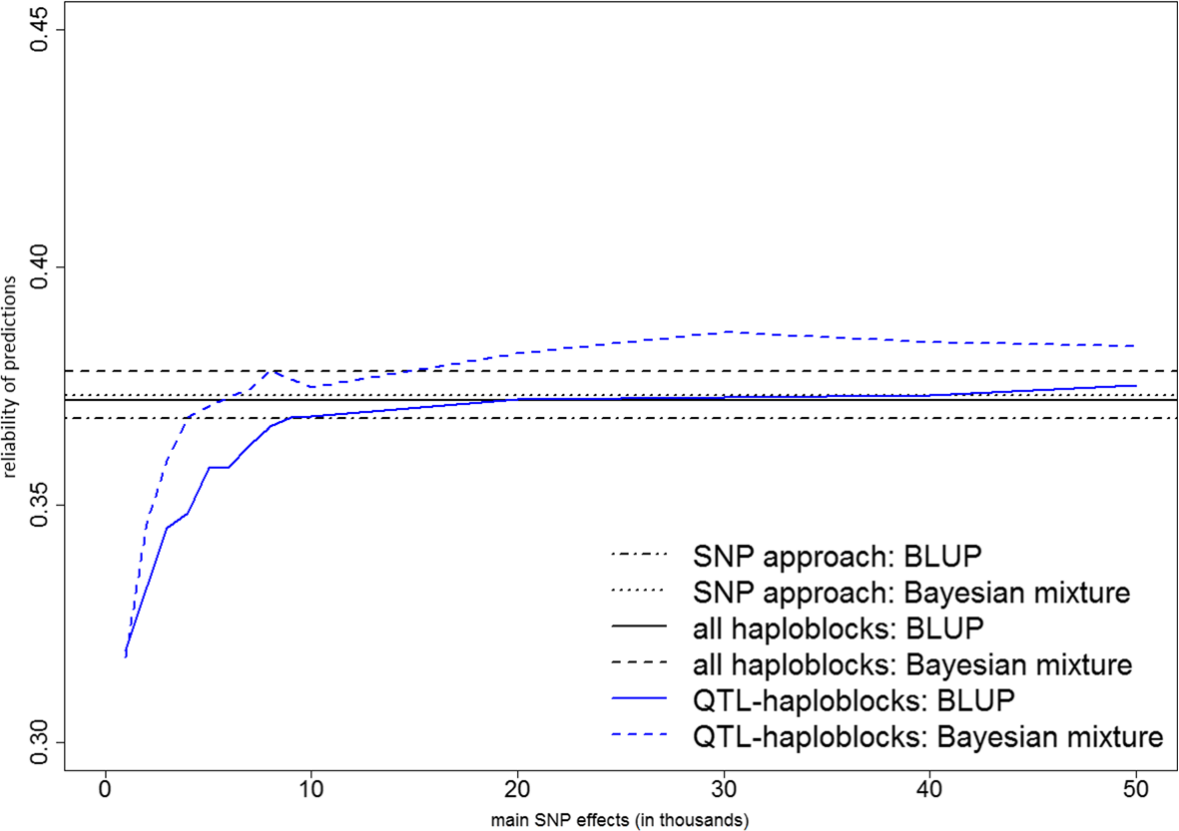
Prediction reliabilities obtained using models with QTL-haploblocks as covariates for mastitis. The values on the x-axis are the number of main SNPs used to select QTL-haploblocks to perform genomic prediction, and the y-axis indicates the reliability of predictions

Figures 1, 2 and 3 present the prediction reliabilities in graphs, for protein yield, fertility and mastitis, respectively. These figures show the fast increase of prediction reliabilities when using up to 10 000 main SNP effects to select QTL-haploblocks. Thereafter, the curves stabilize around the reliabilities obtained by the models that used all haploblocks.

**Fig. 4:**
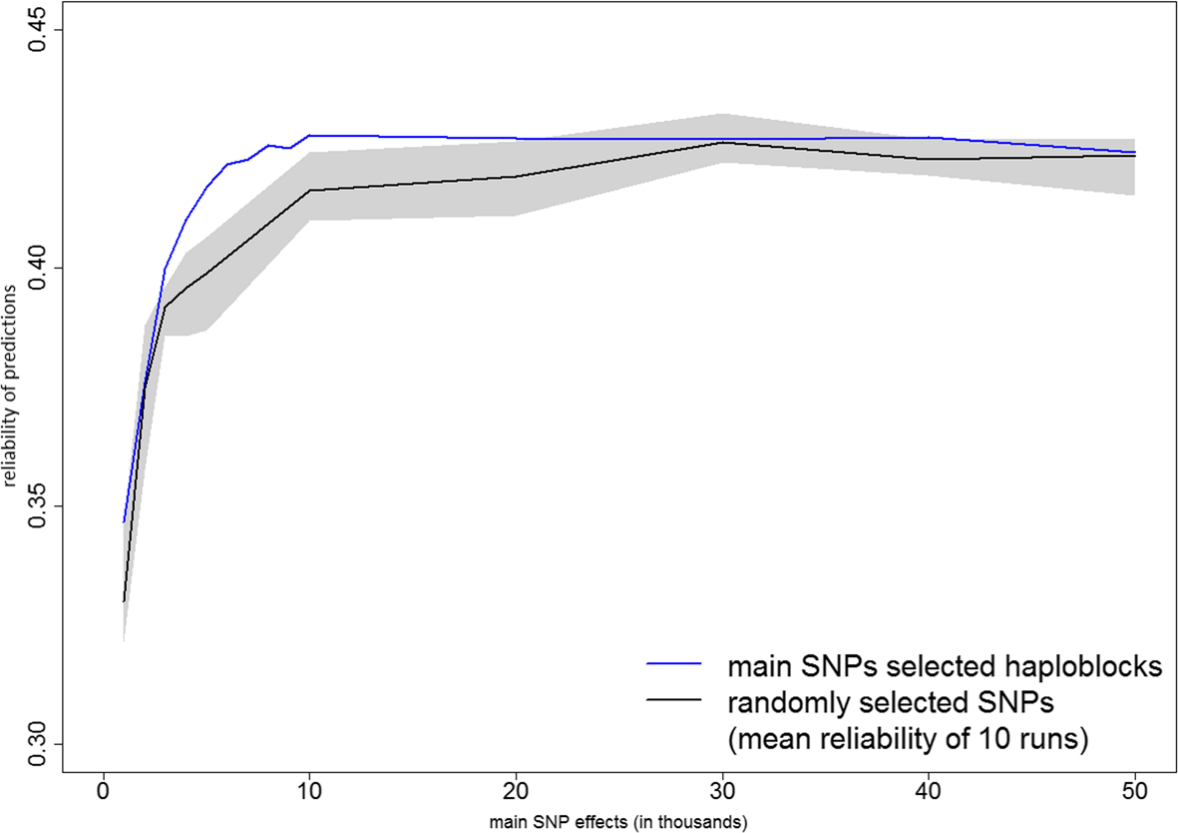
Prediction reliabilities obtained using Bayesian BLUP model with QTL-haploblocks and random selected haploblocks as covariates for protein yield. The values on the x-axis are the number of main SNPs used to select QTL-haploblocks to perform genomic prediction, and the y-axis indicates the reliability of predictions. The grey shaded area shows the range (minimum to maximum prediction reliabilities) and the black lines indicate the mean reliabilities obtained of the models using the randomly selected haploblocks

**Fig. 5:**
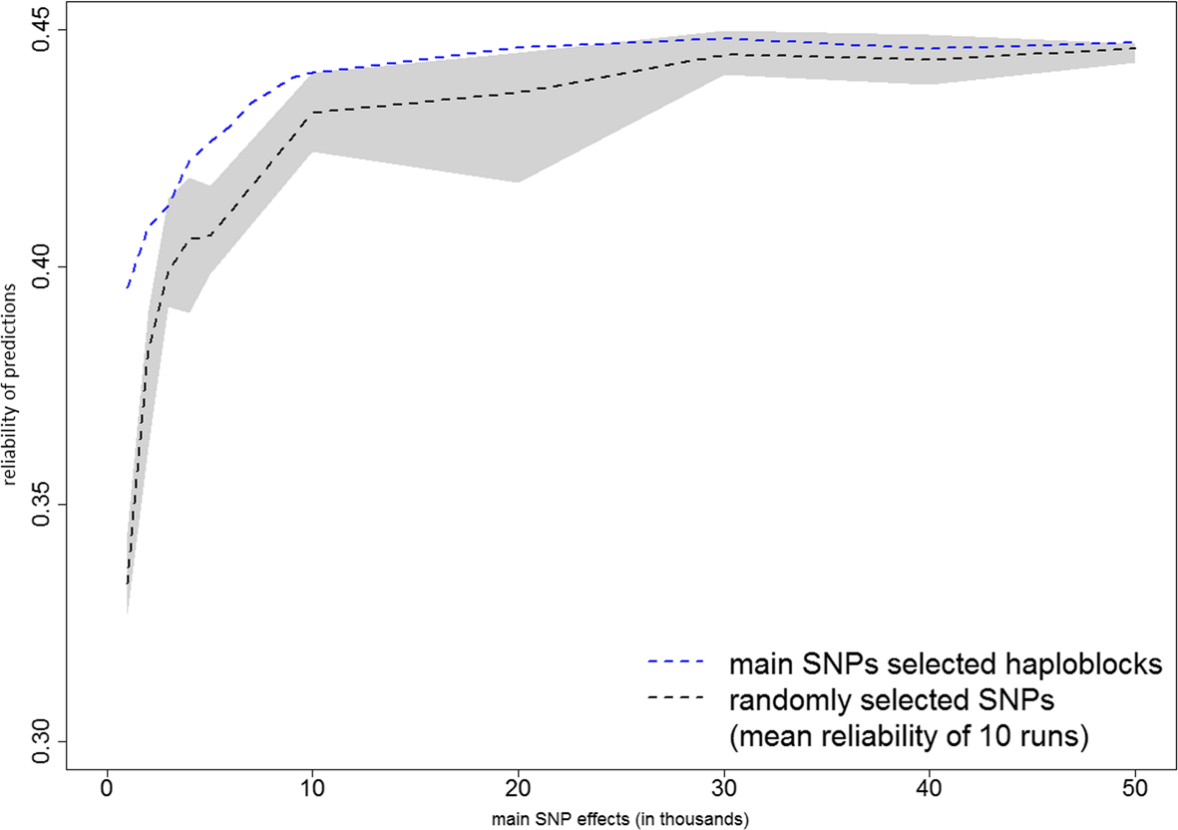
Prediction reliabilities obtained using the Bayesian mixture model with QTL-haploblocks and random selected haploblocks as covariates for protein yield. The values on the x-axis are the number of main SNPs used to select QTL-haploblocks to perform genomic prediction, and the y-axis indicates the reliability of predictions. The grey shaded area shows the range (minimum to maximum prediction reliabilities) and the black lines indicate the mean reliabilities obtained of the models using the randomly selected haploblocks

Figures 4 and 5 compare the results obtained using QTL-haploblocks (blue lines) and randomly selected haploblocks (shaded areas and black lines). Figure 4 shows the results using Bayesian BLUP and Fig. 5 shows the results using the Bayesian mixture model. The random subset of haploblocks was repeated 10 times for each number of SNPs used to select haploblocks (1000 to 50 000), and predictions were performed for each subset. For both Bayesian BLUP and Bayesian mixture models, the mean reliability of the randomly selected haploblocks was lower than those achieved by the QTL-haploblocks, and most of the shaded area is below the blue lines. This confirmed that QTL-haploblocks are better explanatory variables for genomic prediction than haploblocks selected by a random subset of SNPs. It was expected that an advantage of QTL-haploblocks over randomly selected haploblocks would be observed, based on the use of selected individual SNPs for genomic prediction. As shown in Figs. 4 and 5, when a group of individual SNPs were selected based on their estimated effects, the genomic prediction obtained using this group was superior than would be observed if using a randomly selected group of individual SNPs.

Table 5 presents P-values of the two-tailed Hotelling-Williams t-test to verify if the reliabilities obtained using QTL-haploblocks were different from those obtained using all haploblocks. The comparisons were made within each trait and each statistical model. Strictly, it is assumed that if the P-value is greater than 0.05, there is no evidence that the prediction reliabilities are statistically different. However, the closer to 1 the P-value is, the stronger the evidence that the prediction reliabilities are not equal. Table 5 shows that the P-values of the test became higher as the number of main SNPs used to select QTL-haploblocks increased. In general, P-values were high (0.632 to 0.999) when using 20 000 to 50 000 main SNPs, and low (0.063 to 0.268) when using 1000 to 3000 main SNPs.

**Table 5:** P-values for the Hotelling-Williams’ t-statistic to test *H*_0_: the reliability obtained by the model selecting haploblocks is equal to the reliability obtained by the model using all the haploblocks *vs.* *H*_1_: the reliabilities are different, according to number of main SNP effects

Main	Protein			Fertility			Mastitis	
SNPs ^*†*^	BLUP	4 mixture		BLUP	4 mixture		BLUP	4 mixture
1000	0.00	0.00		0.15	0.01		0.00	0.00
2000	0.00	0.00		0.26	0.01		0.03	0.09
3000	0.03	0.01		0.06	0.16		0.14	0.32
4000	0.15	0.05		0.17	0.36		0.19	0.60
5000	0.34	0.10		0.34	0.49		0.42	0.69
6000	0.55	0.16		0.31	0.51		0.43	0.77
7000	0.60	0.30		0.34	0.57		0.59	0.84
8000	0.79	0.41		0.45	0.79		0.74	0.98
9000	0.74	0.54		0.45	0.79		0.82	0.93
10 000	0.91	0.60		0.54	0.96		0.83	0.85
20 000	0.87	0.93		0.82	0.76		0.98	0.82
30 000	0.85	0.91		0.81	0.93		0.99	0.63
40 000	0.88	0.91		0.76	0.99		0.96	0.72
50 000	0.65	0.98		0.86	0.88		0.87	0.75

^*†*^number of highest (absolute) SNP effects used to select haploblocks

**Fig. 6:**
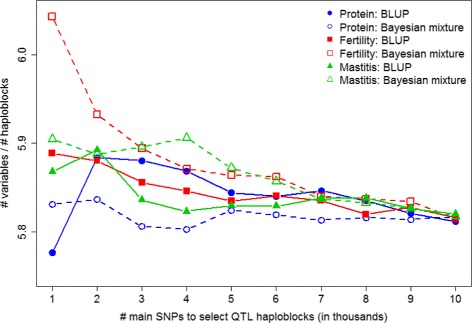
Ratio between total number of haploblock variables used in the prediction models and total number of haploblocks containing the main SNP effects. The values on the x-axis are the number of main SNPs used to select QTL-haploblocks to perform genomic prediction, and the y-axis indicates the ratio (haploblock variables)/haploblocks

One interesting point about the selection of QTL-haploblocks was the relationship between the number of QTL-haploblocks selected by the main SNPs (Table 2) and the number of haploblock variables (Table 3). Figure 6 shows the average number of “alleles” per haploblock, for selection of QTL-haploblocks using 1000 to 10 000 main SNPs. For selection of QTL-haploblocks to predict fertility, the average number of “alleles” per haploblock was greater when using the Bayesian mixture model, than when using Bayesian BLUP. This difference was more accentuated at small numbers of main SNPs (1000 to 2000) to select QTL-haploblocks, and remained almost unchanged up to 6000 main SNPs. For 7000 main SNPs and more, the average number of “alleles” per haploblock became similar, for both the Bayesian BLUP and the Bayesian mixture models. For mastitis, the number of “alleles” per haploblock is also greater when using the Bayesian mixture model up to 6000 main SNPs, except when using 2000 main SNPs, for which the average was slightly greater when using Bayesian BLUP. This difference is more accentuated when using 3000 to 5000 of main SNPs to select QTL-haploblocks. For 7000 main SNPs and more, the difference started to decrease until the ratios converged to the same value. For protein yield, the scenario observed was different, except when using 1000 main SNPs to select QTL-haploblocks, the number of “alleles” per haploblock was greater when using the Bayesian BLUP model, this was most pronounced up to 4000 main SNPs, then converged to the same value.

Table 6 indicates the computing time required to perform the prediction with each model. It was observed that computing time increased according to the increase of main SNPs used to select QTL-haploblocks, hence the increase in predictive variables. It was clear that there was a reduction in computing time, when QTL-haploblocks were used to perform genomic prediction, and this is one relevant benefit of this method.

**Table 6:** Computing time needed to run the genomic prediction models of the three traits using Bayesian BLUP or mixture models, according to number of main SNP effects

Main		Protein			Fertility			Mastitis	
SNP effects		BLUP	4 mixture		BLUP	4 mixture		BLUP	4 mixture
1000		1h 11m	1h 17m		1h 28m	1h 31m		1h 27m	1h 15m
2000		2h 33m	3h 09m		2h 51m	2h 56m		2h 30m	2h 30m
3000		3h 35m	3h 47m		4h 14m	4h 19m		3h 37m	4h 14m
4000		4h 42m	4h 52m		5h 35m	5h 40m		4h 51m	5h 37m
5000		5h 45m	5h 55m		6h 53m	6h 59m		6h 00m	6h 52m
6000		6h 54m	8h 10m		8h 10m	8h 18m		8h 05m	8h 12m
7000		7h 50m	9h 14m		9h 24m	9h 31m		8h 07m	9h 23m
8000		8h 54m	11h 24m		10h 37m	10h 45m		9h 08m	10h 41m
9000		10h 08m	14h 30m		11h 51m	11h 59m		12h 10m	11h 52m
10 000		10h 52m	17h 05m		12h 59m	13h 09m		12h 59m	12h 58m
20 000		19h 59m	19h 56m		23h 44m	23h 50m		23h 38m	27h 42m
30 000		31h 54m	32h 20m		32h 18m	32h 40m		31h 54m	32h 02m
40 000		39h 02m	39h 31m		39h 32m	39h 54m		39h 22m	39h 34m
50 000		45h 16m	45h 46m		45h 51m	46h 11m		45h 22m	45h 44m
492 057		101h 47m	100h 41m		108h 08m	80h 17m		80h 15m	91h 18m

## Discussion

Previous studies have already determined that haploblocks are able to better predict breeding values of economically important traits in dairy cattle, than individual SNPs [3, 4, 7]. Similar to results obtained in [7], the QTL-haploblocks used as predictors in this work may achieve predictions that are more accurate than those using all 492 057 individual SNPs, and as good as those achieved using all 76 062 haploblocks built from the genomic data.

When compared to the predictions with individual SNPs, the QTL-haploblocks were able to achieve higher prediction reliabilities for the three traits, when selected by a range of 20 000 to 50 000 main SNPs. This can be verified in Table 4 and clearly observed in Figs. 1, 2 and 3. This range of 20 000 to 50 000 main SNPs to select QTL-haploblocks was also found to result in prediction reliabilities either equal or very close to those obtained using all haploblocks for protein yield and fertility. In the prediction of mastitis, the reliabilities observed in this range were equal or even greater than those obtained using all haploblocks (up to 0.8 %). This particular result for mastitis was satisfying, taking into account that it is a trait with low heritability, and it is difficult to improve its prediction accuracy using haploblocks [4]. Furthermore, genetic progress is linearly related to accuracy of genetic evaluation. Considering a large dairy cattle population, a small improvement in reliability of predictions is considered important for cattle breeding.

The P-values of the Hotteling-Williams tests in Table 5 were used to compare the results of using only QTL-haploblocks in prediction reliability, with that when using all haploblocks. There was a strong interest in verifying how the increase of variables in the prediction model affected the evidence (P-value) favouring the hypothesis of equal reliabilities. For all the traits, the P-values indicated that the prediction reliabilities using QTL-haploblocks selected by at least 4000 main SNPs (*i.e.* the SNPs with the highest effects) were statistically not different to those obtained using all haploblocks. We could observe, moreover, that while we used 1000 to 10 000 main SNPs to select QTL-haploblocks, the P-values comparing the prediction reliabilities to the full haploblocks model increased regularly. This means that for up to 20 000 main SNPs, the more SNPs we use to select QTL-haploblocks, the stronger becomes our evidence that the prediction reliabilities are not different. In the range of 20 000 to 50 000 main SNPs used to select QTL-haploblocks, the P-values were high (greater than 0.6), suggesting that those models predict as well as or equally well as the model using all haploblocks.

One important feature of prediction models using QTL-haploblocks is the reduction in computing time. Compared to the individual SNP approach, the models with QTL-haploblocks take approximately only 20 to 25 % of the computing time, and 30 to 41 % when compared to using all haploblocks. Thus, although the increase in prediction reliability is small compared to the individual SNP approach (however still very important in cattle breeding), the increase in computational efficiency was considerable. Furthermore, in all our models, we used a MCMC with a fixed length of 50 000 iterations, and the first 20 000 were discarded as burn-in. Because the models that use QTL-haploblocks have significantly less explanatory variables, and because these variables are also less correlated to each other as are individual SNPs, it is expected that the number of MCMC iterations can be reduced. Consequently, a further reduction in computing time can be achieved.

For low or moderate density marker data, the computational gain provided by the use of QTL-haploblocks, from preparation of data and time to run prediction models, may not be relevant. However, the predictions using QTL-haploblocks will be of great importance when it comes to denser marker data, such as whole-genome sequence data. Hence, further studies on genomic prediction using haploblocks and QTL-haploblocks based on LD is a natural next step to evaluate the benefits from these predictors.

## Conclusions

The results from this study suggest that when 20 000 to 50 000 main SNPs were used to select QTL-haploblocks, the use of QTL-haploblocks as predictors is generally sufficient to obtain reliabilities equal or higher than those obtained using all individual SNPs (up to 0.9 % increase for proteinyield, equivalent prediction for fertility and up to 1.3 % increase for mastitis, compared to the individual SNP approach), and similar to those obtained using all haploblocks.

In addition, the method presented here had a positive effect on computing time for prediction models using HD marker data. Compared to the computing time required for the models using all haploblocks, the model using the QTL-haploblocks containing 20 000 to 50 000 main SNPs took on average 40 % of the total time needed and obtained statistically similar results. The computing time for the models using QTL-haploblocks can be further reduced by using less MCMC cycles, since there are less explanatory variables. With denser marker data and whole-genome sequence data, the reduction in computing time would be an important issue in practical genomic prediction.

## References

[1] Meuwissen THE, Hayes BJ, Goddard ME (2001). Prediction of total genetic value using genome-wide dense marker maps. Genetics.

[2] Zondervan KT, Cardon LR (2004). The comples interplay among factors that influence allelic association. Nat Rev Genet.

[3] Villumsen TM, Janss L, Lund MS (2008). The importance of haplotype length and heritability using genomic selection in dairy cattle. J Anim Breed Genet.

[4] Cuyabano BCD, Su G, Lund MS (2014). Genomic prediction of genetic merit using LD-based haplotypes in the Nordic Holstein population. BMC Genomics.

[5] Luan T, Wooliams JA, Ødegård J, Dolezal M, Roman-Ponce SI, Bagnato A (2012). The importance of identity-by-state information for the accuracy of genomic selection. Genet Sel Evol.

[6] Ødegård J, Meuwissen THE (2014). Identity-by-descent genomic selection using selective and sparse genotyping. Genet Sel Evol.

[7] Boichard D, Guillaume F, Baur A, Croiseau P, Rossignol MN, Boscher MY (2012). Genomic selection in French dairy cattle. Anim Prod Sci.

[8] Dekkers JCM, Hospital F (2002). The use of molecular genetics in the improvement of agricultural populations. Nat Rev Genet.

[9] Heffner EL, Sorrels ME, Jannink JL (2009). Genomic selection for crop improvement. Crop Sci.

[10] Matukumalli LK, Lawley CT, Schnabel RD, Taylor JF, Allan MF, Heaton MP (2009). Development and characterization of a high density SNP genotyping assay for cattle. PLoS ONE.

[11] Browning BL, Browning SR (2009). A unified approach to genotype imputation and haplotype-phase inference for large data sets of trios and unrelated individuals. Am J Hum Genet.

[12] Lund MS, de Roos APW, de Vries AG, Druet T, Ducrocq V, Fritz S (2011). A common reference population from four European Holstein populations increases reliability reliability of genomic predictions. Genet Sel Evol.

[13] Su G, Brøndum RF, Ma P, Guldbrandtsen B, Aamand GP, Lund MS (2012). Comparison of genomic predictions using medium-density (∼54,000) and high-density (∼777,000) single nucleotide polymorphism marker panels in Nordic Holstein and Red Dairy Cattle populations. J Dairy Sci.

[14] Jairath L, Dekkers JCM, Schaeffer LR, Liu Z, Burnside EB, Kolstad B (1998). Genetic evaluation for herd life in Canada. J Dairy Sci.

[15] Schaeffer LR (2001). Multiple trait international bull comparisons. Livest Prod Sci.

[16] Garrick DJ, Taylor JF, Fernando RL (2008). Deregressing estimated breeding values and weighting information for genomic regression analyses. Genet Sel Evol.

[17] Hill WG, Robertson A (1968). Linkage disequilibrium in finite populations. Theor Appl Genet.

[18] Hill WG (1981). Estimation of effective population size from data on linkage disequilibrium. Genet Res.

[19] Gabriel SB, Schattner SF, Nguyen H, Moore JM, Roy J (2002). The structure of haplotype blocks in the human genome. Science.

[20] BayZ Manual, version 2.04. 2013. ed. http://www.bayz.biz/.

[21] VanRaden PM (2008). Efficient methods to compute genomic predictions. J Dairy Sci.

[22] Su G, Madsen P, Nielsen US, Mäntysaari EA, Aamand GP, Christensen OF (2012). Genomic prediction for Nordic Red Cattle using one-step and selection index blending. J Dairy Sci.

[23] Gao H, Su G, Janss L, Zhang Y, Lund MS (2013). Model comparison on genomic predictions using high-density markers for different groups of bulls in the Nordic Holstein population. J Dairy Sci.

[24] George EI, McCulloch RE (1993). Variable selection via Gibbs sampling. J Am Statist Assoc.

[25] Meuwissen THE (2009). Accuracy of breeding values of ‘unrelated’ individuals predicted by dense SNP genotyping. Genet Sel Evol.

[26] Hotelling H (1940). The selection of variates for use in predictions with some comments on the problem of nuisance parameters. Ann Math Stat.

[27] Williams EJ (1959). Regression Analysis.

[28] R Core Team (2014). R. A language and environment for statistical computing.

